# Simultaneous Estimation of Quercetin and *trans*-Resveratrol in *Cissus quadrangularis* Extract in Rat Serum Using Validated LC-MS/MS Method: Application to Pharmacokinetic and Stability Studies

**DOI:** 10.3390/molecules28124656

**Published:** 2023-06-08

**Authors:** Shailesh D. Dadge, Anees A. Syed, Athar Husain, Guru R. Valicherla, Jiaur R. Gayen

**Affiliations:** 1 Pharmaceutics & Pharmacokinetics Division, CSIR-Central Drug Research Institute (CSIR-CDRI), Sitapur Road, Lucknow 226031, India; shailesh.dadge@gmail.com (S.D.D.);; 2 Academy of Scientific and Innovative Research, Ghaziabad 201002, India; 3 Pharmacology Division, CSIR-Central Drug Research Institute (CSIR-CDRI), Lucknow 226031, India

**Keywords:** *Cissus quadrangularis*, LC-MS/MS, quercetin, pharmacokinetics, *trans*-resveratrol, validation

## Abstract

*Cissus quadrangularis* is a nutrient-rich plant with a history of use in traditional medicine. It boasts a diverse range of polyphenols, including quercetin, resveratrol, β-sitosterol, myricetin, and other compounds. We developed and validated a sensitive LC-MS/MS method to quantify quercetin and *t*-res biomarkers in rat serum and applied this method to pharmacokinetic and stability studies. The mass spectrometer was set to negative ionization mode for the quantification of quercetin and *t*-res. Phenomenex Luna (C18(2), 100 A, 75 × 4.6 mm, 3 µ) column was utilized to separate the analytes using an isocratic mobile phase consisting of methanol and 0.1% formic acid in water (82:18). Validation of the method was performed using various parameters, including linearity, specificity, accuracy, stability, intra-day, inter-day precision, and the matrix effect. There was no observed significant endogenous interference from the blank serum. The analysis was completed within 5.0 min for each run, and the lower limit of quantification was 5 ng/mL. The calibration curves showed a linear range with a high correlation coefficient (r^2^ > 0.99). The precision for intra- and inter-day assays showed relative standard deviations from 3.32% to 8.86% and 4.35% to 9.61%, respectively. The analytes in rat serum were stable during bench-top, freeze-thaw, and autosampler (−4 °C) stability studies. After oral administration, the analytes showed rapid absorption but underwent metabolism in rat liver microsomes despite being stable in simulated gastric and intestinal fluids. Intragastric administration resulted in higher absorption of quercetin and *t*-res, with greater C_max_, shorter half-life, and improved elimination. No prior research has been conducted on the oral pharmacokinetics and stability of anti-diabetic compounds in the Ethanolic extract of *Cissus quadrangularis* EECQ, making this the first report. Our findings can provide the knowledge of EECQ’s bioanalysis and pharmacokinetic properties which is useful for future clinical trials.

## 1. Introduction

In 2015, WHO estimated that 70–80% of people worldwide rely on herbal remedies from plants for healthcare [1]. Quercetin and resveratrol are attracting a lot of attention as polyphenols due to their multiple biological effects, potential therapeutic value, and abundance in various foods [2]. Plant-based medicines, preferred by patients for promoting well-being, contain bioactive compounds sought after for their therapeutic properties over synthetic alternatives [3]. The broad usage assumes their safety and effectiveness for extended public use *Cissus quadrangularis*, a tropical climbing shrub, is native to India, Malaysia, Sri Lanka, and West Africa [4]. It exhibits hypoglycemic properties [5] and anti-glycation properties [6] due to various compounds, including phytosterols, flavonoids, steroidal substances, and triterpenoids [7]. Moreover, studies indicate that *Cissus quadrangularis* powder contains diverse nutrients, including protein. It contains fat, fiber, and various minerals. *Cissus quadrangularis* is consumed as food in Nigeria [8]. Extracts from *Cissus quadrangularis* have been used for managing osteoporosis, menstrual disorders, pain relief, wounds, burns, and metabolic/cardiovascular disorders [9]. Previously, our group found that *Cissus quadrangularis* treatment improved diabetic nephropathy [10], diabetic cardiomyopathy [11], and insulin resistance in menopausal rats [12], and hypertension in SHR rats [13]. *Cissus quadrangularis* treatment improved metabolic parameters in rats on a high-fat diet. Studies have explored the pharmacological properties of *Cissus quadrangularis*, including anti-inflammatory [14,15] analgesic [15,16], antimicrobial [17], and antioxidant effects [18]. The plant’s medicinal properties are linked to bioactive compounds such as resveratrol, piceatannol, pallidol, parthenocissine A, stilbene derivatives, and quadrangularins A, B, and C [19]. The plant contains various constituents, including quercetin, quercitrin, β-sitosterol, and other compounds such as 6-O-[2,3-dimethoxy]-trans-cinnamoyl catalpol and 6-O-meta-methoxy-benzoyl catalpol [20]. *Cissus quadrangularis* is used to treat various health conditions [18]. *Cissus quadrangularis* has been used in Ayurveda and Unani medicine for various purposes, including digestive, laxative, aphrodisiac, and bone fracture healing properties [21].

Recent studies have concentrated on investigating the combination of two or more flavonoids to enhance the pharmacological impact [22]. Quercetin and *t*-res are two polyphenols found naturally that have the ability to promote health [2]. Polyphenols have low bioavailability and quickly convert to phase II conjugates, resulting in low levels of unmodified compounds in the bloodstream. Current efforts involve developing pro-drugs, formulations, and macromolecular vectors such as liposomes and nanoparticles to improve absorption [23]. Various techniques have been established to evaluate these polyphenols in different biological samples, such as plasma, urine, and tissues [22]. Most pharmacokinetic studies have focused on measuring polyphenol levels in plasma or serum [24]. Plasma analysis may not provide accurate information on overall circulating levels of quercetin [25] and *t*-res [26] as these polyphenols can bind to blood cells, proteins, and DNA, forming a “reserve” that can replace molecules eliminated through metabolism and excretion.

Pharmacokinetic studies clarify how highly pharmacological potential molecules are distributed and available in crude extracts [27] or as isolated metabolites [28]. Only a few studies have used small samples of 30–200 µL of serum or plasma collected via capillary microsampling [29]. HPLC-MS/MS was used in prior research to measure analytes in rat plasma for the pharmacokinetics of polymethoxylated flavones [30], phenolic acids, and flavonoids [31]. HPLC-MS/MS is also used in pharmacokinetic studies of organic crude extracts [27]. To quantify specific flavonoids rather than the total molecules of the extract. Crude extracts are the traditional form of administration for herbal medicines. The predicted efficacy of herbal medicines may be due to favorable drug metabolism and the synergistic effect between multiple active compounds, despite their low abundance. To investigate the pharmacokinetics and metabolic study of two active phytoconstituents in *Cissus quadrangularis* extract, a pilot pharmacokinetic study in rats was conducted, which utilized a simple and sensitive LC-MS/MS bioanalytical method. Studying the pharmacokinetic parameters of flavonoids is crucial in the preclinical stage. Oral administration of single flavonoids and the combination of quercetin and *t*-res need to be compared to explain their bioavailability. This comparison has not been reported in the literature so far. However, the pharmacokinetic properties and stability of quercetin and *t*-res in *Cissus quadrangularis* extract have not been fully elucidated. Analytical reports on this topic have limitations, such as the type of solvent system used, whether a gradient or isocratic process was employed, sample analysis time, and the adsorbent system utilized method for separating *t*-res markers has a lengthy analysis time of 30 min, a poor baseline, and utilizes a gradient elution mode [32]. A method for the analysis and separation of *t*-res and its metabolite using mass spectrometry and HPLC was reported, but the method has a relatively long runtime of 19 min [33]. A recently published HPLC-PDA method for the separation of quercetin and *t*-res has been reported, but the analysis time is relatively long, approximately 24 min [34].

Our method enables the simultaneous analysis of quercetin and *t*-res in a single run. While individual quantification methods for these compounds have been reported before, the simultaneous analysis of both compounds in a single method is novel and offers several advantages. It saves time, reduces sample preparation, and allows for a more comprehensive assessment of these compounds in complex matrices. The method exhibits significantly improved sensitivity compared to previously reported methods. By employing a novel extraction technique and optimizing the chromatographic conditions, we achieved lower detection limits and enhanced quantification accuracy for both quercetin and *t*-res. This increased sensitivity allows for the reliable analysis of trace amounts of these compounds in complex matrices. The method has been specifically optimized and validated for the quantification of quercetin and *t*-res in a specific sample matrix, which is a significant advancement compared to previous methods. By considering the characteristics and challenges associated with our target sample matrix, we have tailored the method to ensure accurate and reliable quantification in real-world applications. The developed method demonstrates improved sensitivity and selectivity for the detection and quantification of quercetin and *t*-res. We have optimized various parameters, such as mobile phase composition, column type, and detection wavelength, to achieve superior analytical performance compared to previously reported methods. This enhancement allows for more precise and reliable quantification of these compounds, even at lower concentrations. This method exhibits robustness and high reproducibility, as evidenced by rigorous validation tests. We thoroughly investigated the method’s precision, accuracy, linearity, and stability under various conditions. The obtained results demonstrate the reliability and repeatability of our method, making it suitable for routine analysis in research and industrial laboratories. Finally, we successfully applied our developed method to analyze quercetin and trans-resveratrol in complex matrices such as biological samples or plant extracts. By addressing the challenges associated with these matrices, including potential interferences and sample matrix effects, our method provides a valuable tool for the accurate quantification of these compounds in real-world samples.

The novelty of the manuscript is to develop and validate the simple, sensitive, rapid LC-MS/MS method for the simultaneous quantification of quercetin and *t*-res in rat serum and apply this method to investigate the in vitro and in vivo pharmacokinetic properties of quercetin and *t*-res in rats. This study aims to develop and validate the liquid chromatography tandem mass spectrometry (LC-MS/MS) method for simultaneous quantification of quercetin and *t*-res levels in rat serum. The validated method will be applied to the pharmacokinetic and stability studies of these compounds. The validated LC-MS/MS method for the estimation of quercetin and *t*-res from *Cissus quadrangularis* extract in rat serum is essential to understand their pharmacokinetic properties. The results of this study will provide important information for the development of *Cissus quadrangularis* extract-based formulations and the therapeutic potential of *Cissus quadrangularis* extract in animal models and humans for the treatment of various diseases.

## 2. Results

### 2.1. Mass Spectrometry Conditions

**Figure 1:**
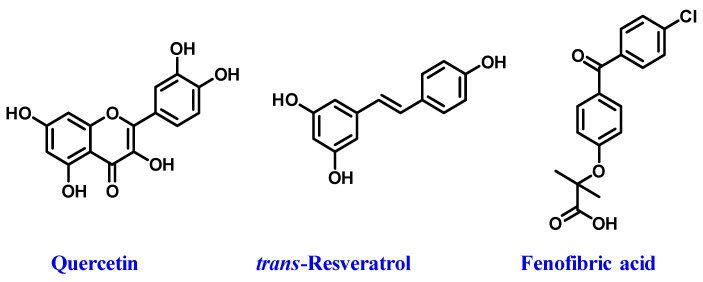
Chemical structure of *quercetin*, *t*-*re*s from *Cissus quadrangularis* stem extract and fenofibric acid (IS).

**Figure 2:**
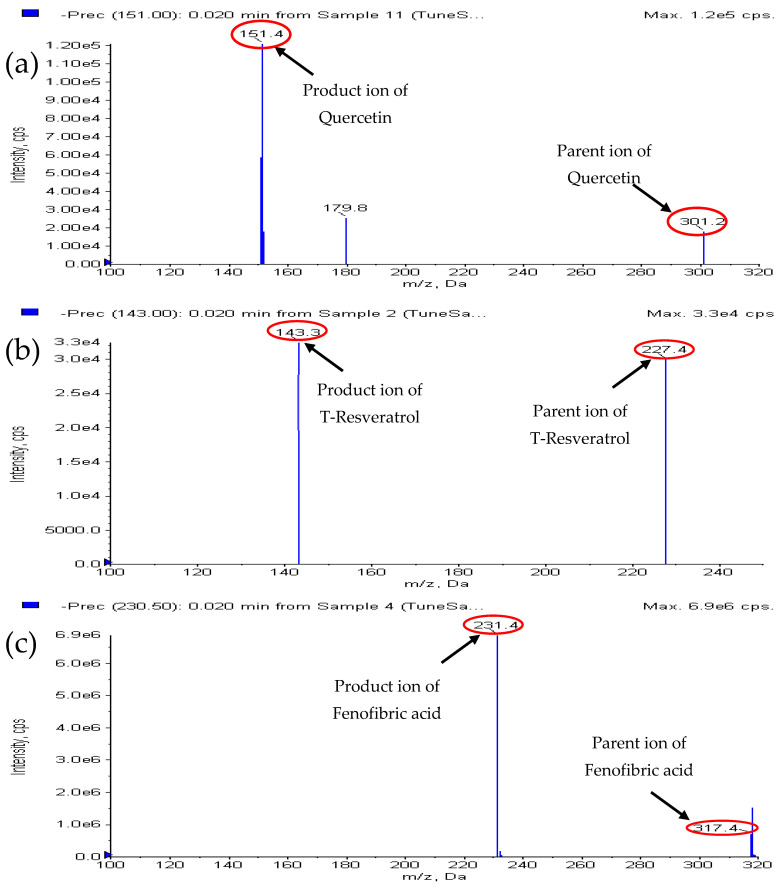
MS/MS spectra of (**a**) Quercetin, (**b**) *t*-res, and (**c**) ffa (I.S).

The quantification of quercetin and *t*-res was done using an ESI source-equipped (Applied Biosystems, MDS Sciex Toronto, Toronto, ON, Canada) AB Sciex 4000 QTRAP mass spectrometer. To optimize quercetin and *t*-res, and I.S. parameters dependent on the source and compound, an infusion pump was used for continuous direct infusion of a neat solution. Quercetin and *t*-res and I.S. exhibited higher intensity when analyzed in negative ionization mode, which was also utilized alongside positive mode. Figure 1 illustrates the chemical structures of quercetin, *t*-res, and ffa (I.S.). Table 1 demonstrates the optimized compound and source parameters for quercetin, *t*-res, and I.S., achieved in negative mode. Quercetin, *t*-res and ffa (I.S.) mass spectra exhibited [M-H]^−1^ deprotonated molecular ion peaks at *m*/*z* 301, 227, and 317, respectively. Figure 2 clearly showed the mass fragmentation patterns of quercetin, *t*-res, and ffa (I.S.). The MRM transitions were selected for quantification of each analyte as follows: *m*/*z* 301 *>* 151 for quercetin, 227 *>* 143 for *t*-res, and 317 *>* 230.9 for ffa (I.S.).

### 2.2. Liquid Chromatography Conditions

After the selection of MS parameters, LC conditions were optimized by changing the mobile phase composition, buffer pH, and flow rate to achieve improved sensitivity and good peak shape. Numerous columns were tested to enhance resolution, selectivity, and analysis speed. Phenomenex Luna column (C18(2), 100A, 75 × 4.6 mm, 3 μ) was found to be suitable for the separation of quercetin *t*-res, and I.S. using an isocratic mobile phase comprising 82:18 (*v*/*v*) methanol:0.1% formic acid in TDW with a flow rate of 0.5 mL/min. The analysis run time was 5 min. After evaluating the multiple compounds for their similarity in chromatographic retention, ionization efficiency, and extraction behavior with quercetin, *t*-res, and ffa was chosen as the internal standard.

### 2.3. Serum Samples Processing

The combination of protein precipitation (PP) and LLE methods yielded satisfactory recovery without any noticeable matrix effect. MeOH was used for PP and ethyl acetate was used for LLE. Formic acid containing methanol was used to increase the extraction efficiency. The acidified methanol was used to suppress protein adsorption and promote the release of analytes and I.S from proteins [35]. The β-glucuronidase enzyme was used to hydrolyze the glucuronide conjugates of analytes. It is easier to quantify the free form of an analyte, rather than the glucuronide conjugate form [36].

### 2.4. Method Validation

#### 2.4.1. Selectivity and Specificity

**Figure 3:**
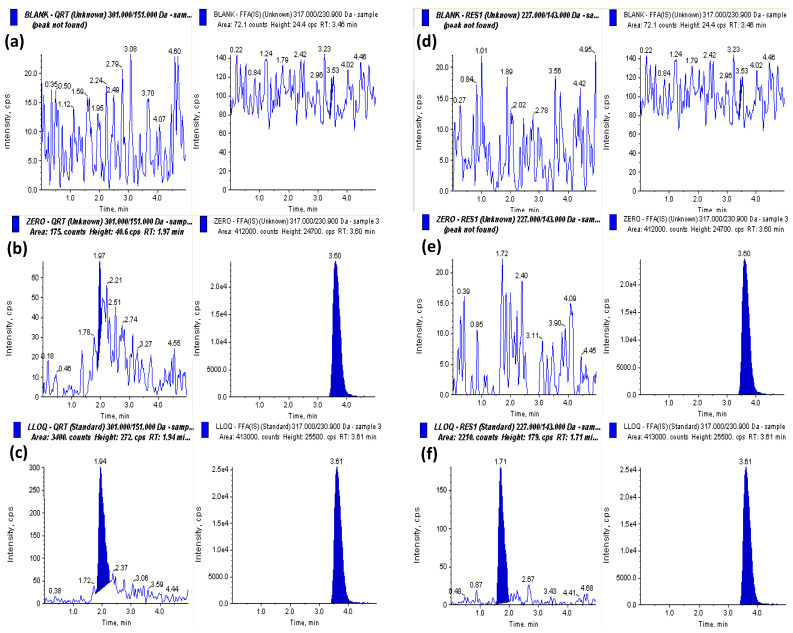
MRM chromatograms of blank plasma, zero sample spiked with IS, and, sample spiked with LLOQ (5 ng/mL) and I.S of (**a**) blank, (**b**) zero, and (**c**) LLOQ spiked with I.S. for quercetin (**d**) blank, (**e**) zero, and (**f**) LLOQ (5 ng/mL) spiked with IS for *t*-res.

No significant chromatographic interferences were detected from the rat serum at the retention times of both analytes and I.S. The optimal LC-MS/MS conditions ensured the selectivity and specificity for both analytes. Figure 3 displays the MRM chromatograms of blank samples, zero samples spiked with I.S, and LLOQ samples spiked with I.S for the analytes.

#### 2.4.2. Linearity and Sensitivity

Table 2 shows the range of linearity, sensitivity, regression analysis, and correlation coefficient values for both analytes in rat serum. LLOQ for the developed LC-MS/MS method was observed as 5 ng/mL for both quercetin and *t*-res. The signal-to-noise ratio for quercetin and *t*-res was found to be 58.2 ≥ 10:1, indicating a robust and reliable quantification limit. The LOD was determined to be 1 ng/mL with a signal-to-noise ratio of 32.7 ≥ 3:1. The accuracy and precision of the back-calculated CS met the acceptance criteria.

#### 2.4.3. Accuracy, Precision, and Carryover Effect

Table 3 shows the accuracy and precision data for intra-day and inter-day assays of quercetin and *t*-res in rat serum at four QC levels. The accuracy (%) varied from 92.77 to 107.39 for both intra-day and inter-day measurements. At four QC levels, RSD (%) ranges from 3.32 to 9.61 as shown in Table 3. Accuracy and precision data were found to be within acceptable limits. The absence of Quercetin, *t*-res, and I.S chromatographic peaks in blank samples injected after the ULOQ sample, indicates that there was no observed carry-over effect.

#### 2.4.4. Dilution Integrity, Recovery and Matrix Effect

The accuracy of dilution integrity samples was found to be 100.72 ± 1.61 for quercetin and 104.03 ± 4.20 for *t*-res. Table 4 shows the extraction recoveries of quercetin and *t*-res with mean values ranging from 80.52% to 87.96% at three concentration levels. The matrix effect varied from 91.40% to 106.41% for both analytes.

#### 2.4.5. Stability

The accuracy and precision data quercetin and *t*-res from the autosampler, benchtop, freeze-thaw, and long-term stability studies as illustrated in Table 5. Both quercetin and *t*-res were stable in rat serum at variable storage conditions commonly encountered during sample processing. The accuracy and precision were found within acceptable limits for stability studies.

#### 2.4.6. Stability of Quercetin and *t*-res in Simulated Gastrointestinal Fluids

**Figure 4:**
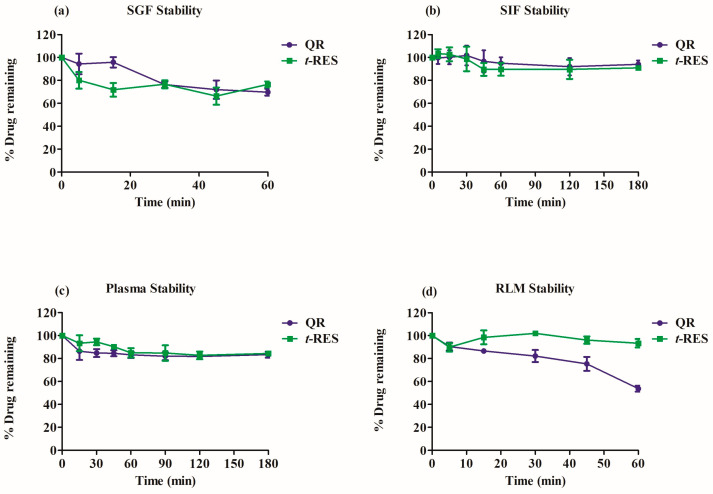
Stability of quercetin and *t*-res in (**a**) simulated gastric fluid, (**b**) simulated intestinal fluid, (**c**) rat plasma, and (**d**) RLM. Data are represented as mean ± SD, n = 3.

Figure 4a,b displays the stability data of quercetin and *t*-res in SGF and SIF. Moderate instability was observed in SGF for quercetin and *t*-res and these compounds were found to be stable in SIF.

#### 2.4.7. Stability Studies in Plasma

Compounds that degrade quickly in plasma can fail to produce therapeutic effects. Plasma stability is a crucial factor in early drug development. Plasma enzyme hydrolysis significantly alters drug bioavailability [37]. Esterase and deamidases are hydrolytic enzymes found in plasma along with many other enzymes [38]. Therefore, the assessment of compound stability in plasma is necessary. The % remaining of quercetin and t-res were found to be 83.43 and 84.43%, respectively at 3 h in rat plasma. quercetin and t-res stability in rat plasma suggests that these compounds would be stable in systemic circulation [39].

#### 2.4.8. Stability Study in Rat Liver Microsome

Figure 4d shows the metabolic stability data of quercetin and t-res in RLM. Liver and intestinal microsomes significantly contribute to the clearance of orally administered drugs via metabolism [40]. Cytochrome P450s are the most significant group of enzymes and have a crucial role in drug metabolism [41]. The % remaining of Quercetin was found to be 53.78, which indicates that phase I metabolism occurred in RLM as shown in Figure 4d. There was no phase-1 metabolism observed for t-res in RLM.

#### 2.4.9. In-Vivo Pharmacokinetic Study in Rats

**Figure 5:**
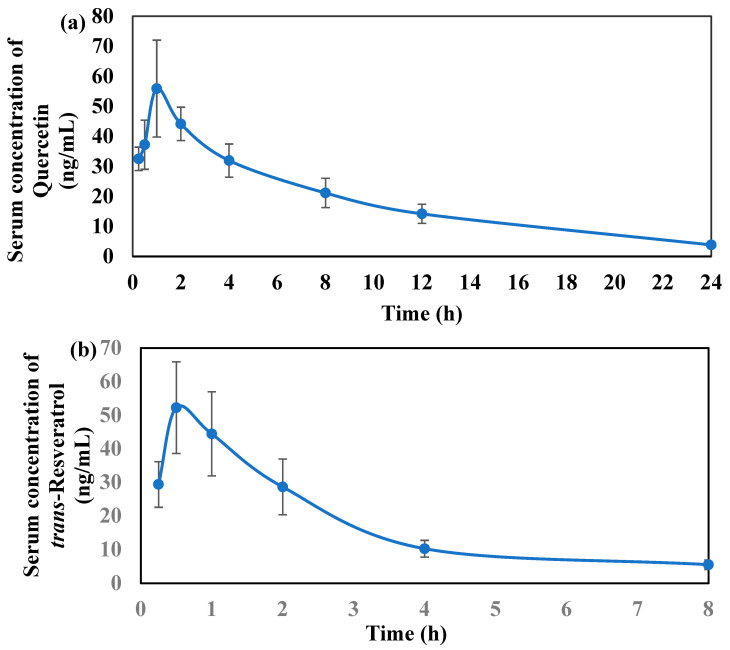
Average serum concentration vs. time profile of (**a**) Quercetin (**b**) *t*-res after oral administration of EECQ at 400 mg/Kg.

Table 6 listed the pharmacokinetics parameters of quercetin and t-res, and Figure 5a,b shows the serum concentration vs. time profiles of quercetin and t-res from EECQ administered orally in male SD rats. The C_max_ of quercetin and t-res were found to be 57.65 ± 14.28 and 56.35 ± 13.14 ng/mL, respectively. The observed T_max_ for quercetin and t-res was 1.17 ± 0.41 and 0.58 ± 0.20 h, respectively. These results indicate that quercetin and t-res showed rapid absorption in oral administration [42]. For quercetin and t-res, AUC_(0–∞)_ was computed as 471.23 ± 51.49 and 156.57 ± 21.09 h*ng/mL, and AUC_(0–t)_ were 447.06 ± 47.05 and 144.83 ± 20.76 (h*ng/mL). This suggests that the appropriate sample duration was selected with the projected terminal half-life [43]. The observed t_1/2_ of quercetin and t-res was 4.91 ± 0.69 and 56.35 ± 13.14 h, respectively, while their CL/F was 851.55 ± 89.69 and 2593.43 ± 346.71 L/h/Kg, respectively [44]. The V_d_ of quercetin and t-res was significantly higher compared to the total blood volume of rats (0.054 L/Kg) and the total water volume of rats (0.668 L/Kg). This indicates that quercetin and t-res have high tissue distribution in rats. quercetin and t-res showed rapid oral absorption with high C_max_, shorter half-life, and high volume of distribution.

## 3. Discussion

The optimization of instrument parameters, chromatographic conditions, mobile phase composition, and column selection was involved in the LC-MS/MS method development to ensure accuracy, sensitivity, and reproducibility. Various solvents and buffers with different pH levels were tried to achieve good resolution of the analyte and I.S. Flow rate and mobile phase composition were modified using various columns to achieve good peak shapes for the analytes and I.S. The Phenomenex Luna column was identified as the optimal choice for achieving high chromatographic resolution using methanol and 0.1% formic acid in TDW (82:18 v/v) at a flow rate of 0.8 mL/min. FFA was chosen as an I.S due to its similar extraction behavior and chromatographic retention with quercetin and t-res. The parameters mentioned above were used to validate and analyze the stability samples of rat serum, and were compared to a previously reported method. Analytical reports on this topic have limitations, such as the type of solvent system used, whether a gradient or isocratic process was employed, sample analysis time, and the adsorbent system utilized. The method for separating t-res markers has a lengthy analysis time of 30 min, a poor baseline, and utilizes a gradient elution mode [32]. A method for the analysis and separation of t-res and its metabolite using mass spectrometry and HPLC was reported, but the method has a relatively long runtime of 19 min. A recently published HPLC-PDA method for the separation of quercetin and t-res has been reported, but the analysis time is relatively long, approximately 24 min [34]. Our study developed and validated a bioanalytical method to quantify quercetin and t-res which are the main phytoconstituents from a commercial Cissus quadrangularis extract in serum. This is different from earlier research that only looked at β-sitosterol, α-amyrin and β-amyrin [45]. We utilized the validated LC-MS/MS method to quantify the serum concentrations of two chemical constituents of EECQ, such as quercetin and t-res. In vitro techniques were used to investigate the stability of quercetin and t-res components in simulated gastric fluid, intestinal fluid, plasma matrices, and microsomal stability in rat liver. Our in vitro results indicated that these constituents were stable in SIF and plasma, but only moderately stable in SGF, with 76% of the compound remaining after 30 min. In vitro stability studies revealed t-res stability in various conditions, which might explain the high plasma levels (C_max_ and AUC values) observed in the pharmacokinetic study. Quercetin showed over 50% phase I metabolism in RLM within 60 min, while no phase I metabolism was observed for t-res in RLM. The results imply that the confirmed bioanalytical technique could be valuable for forthcoming clinical pharmacokinetic studies of EECQ supplements in humans. This article describes a rapid, sensitive, and simple method for concurrently measuring quercetin and t-res in rat serum. The method was used to study the pharmacokinetics of Ethanolic extract of Cissus quadrangularis in rats. The results of the in-vivo pharmacokinetic study in rats provide important information on the pharmacokinetic characteristics of quercetin and t-res in serum after oral administration of EECQ extract. Previous studies found free t-res when higher amounts (>25 mg) were administered [46]. Recently, Meng-xiang Su et al. (2011) reported a pharmacokinetic study of t-resveratrol in rat plasma (0.6 mg/kg) of two different formulation by LC-MS/MS Results revealed that upon oral administration of t-resveratrol T_max_ values were 19 ± 9 min and 110 ± 15 min, C_max_ (23 ± 8 ng mL^−1^), half-life was 36 ± 6 min, e AUC_(0–t)_ values were 2835 ± 1655 min ng mL^−1^ [47]. Dinesh Kumar V et al. (2014) reported LC-ESI-MS/MS analysis of quercetin in rat plasma at a dose of 25 mg/kg followed by oral administration C_max_ was found to be 369.20 at T_max_ of 4.00, plasma half-life was 4.96 h, AUC_(0–t)_ was 3283.75 (ng h/mL) [48]. Results show that quercetin and t-res are rapidly absorbed, as evidenced by their high C_max_ and short T_max._ The results show that quercetin and t-res are rapidly absorbed, as evidenced by their high C_max_ and short T_max._ The AUC_(0–∞)_ values suggest that the sample duration and the projected terminal half-life were appropriately selected. The observed half-lives of quercetin and t-res were significantly different, with t-res exhibiting a much longer half-life. The higher clearance of t-res compared to quercetin may suggest that t-res undergoes high elimination. The apparent volume of distribution of both compounds indicates that they are effectively distributed outside the vascular compartment, possibly in tissues. This study provides valuable information for further pharmacokintic and toxicokinetic studies on EECQ extract and its components, quercetin, and t-res.

## 4. Materials and Methods

### 4.1. Chemicals

Analytical grade chemicals and reagents were used in this analysis including Quercetin (purity ≥ 95%), *trans*-resveratrol (purity ≥ 98%), fenofibric acid (FFA, purity ≥ 98%), and β-glucuronidase from Helix pomatia (Type H-1) (partially purified) purchased from Sigma-Aldrich (St. Louis, MO, USA). LCMS grade methanol (MeOH) and acetonitrile (ACN) purchased from JT Baker (Radnor, PA, USA) were used. Ethyl acetate from Merck Life Sciences. Pvt. Ltd. (Mumbai, India), and formic acid and ammonium acetate from Sisco Research Laboratories (SRL) Pvt. Ltd. (Mumbai, India).

### 4.2. Animals

Male Sprague Dawley (SD) rats were obtained from the National Laboratory Animal Center at CSIR-Central Drug Research Institute (CSIR-CDRI), Lucknow, India. Animals were acclimatized for a week before conducting the experiments. Animal experiments were performed in compliance with the approved Institutional Animal Ethics Committee protocol (IAEC/2018/91). Fresh drug-free serum was collected from male SD rats, and euthanasia was performed after the completion of the study.

### 4.3. LC-MS/MS Analysis

For LC separation, a Shimadzu Ultra-Fast Liquid Chromatography system (Kyoto, Japan) consisting of an LC-20AD pump, DGU-20A degasser, SIL-HTc Autosampler, and CTO-20AC column oven was used. For mass spectrometric detection, an AB Sciex 4000 QTRAP mass spectrometer (Applied Biosystems/MDS Sciex, Toronto, ON, Canada) with an electrospray ionization (ESI) source was employed. Analyst software (version 1.6.1) was utilized to obtain, quantify, and analyze the samples. Phenomenex Luna (C18(2), 100 A, 75 × 4.6 mm, 3 µ) column was utilized to separate the quercetin, *t*-res, and ffa (I.S.) with the isocratic condition of methanol and 0.1% formic acid in triple-distilled water (82:18 *v*/*v*) as the mobile phase at a flow rate of 0.8 mL/min. The run time was 5 min and the injection volume was 10 µL for each sample.

LC-MS/MS analysis was performed in negative ionization mode as described in Table 1. The compound’s source parameters were determined, including an ion spray voltage of 4500 V. The curtain gas was set to 25, and the CAD gas was set to medium. The temperature was maintained at 400 °C, while the ion source gases (GS1 and GS2) were used in a 50:50 ratio.

### 4.4. Characterization of Cissus Quadrangularis Extract

The stem bark of Cissus quadrangularis was procured from the vendor Haridass Aggarwal & Sons, Navi Mumbai, India. The authentication of the plant material was carried out by the Botany Division, CSIR-Central Drug Research Institute (CSIR-CDRI), Lucknow, India, vide voucher specimen number 25,283. The stem of *Cissus quadrangularis* was dried and powdered, then extracted with 95% ethanol by the percolation method. The resulting extract was filtered, concentrated by rotavapor at 40 °C, and yielded 200.0 g. The stem bark extract was then characterized, and quantified using LC-MS/MS analysis as described in a previously published paper [49].

### 4.5. Standard Solutions, Calibration Curve Quality Control (QC) Samples

The primary stock solutions of quercetin and *t*-res (1 mg/mL) were prepared separately in DMSO. Working solutions were prepared by serially diluting the primary stocks in methanol to achieve calibration range (CS; 50–5000 ng/mL) and quality control concentrations (QC; 300, 2030, and 3750 ng/mL). CS/QCs samples were prepared by spiking 10 μL of working solution into the corresponding 90 μL of rat serum. The final concentrations of CS were ranging from 5–500 ng/mL and the QC concentrations were 30, 203, and 375 ng/mL.

### 4.6. Serum Sample Extraction Procedure

Liquid-liquid extraction was employed for processing samples of quercetin and *t*-res in rat serum. CS/QC samples were prepared using 10 μL of sample and 90 μL of control serum or 100 μL serum (in vivo) samples were treated with 100 μL of 1 mg/mL β-glucuronidase enzyme in 100 mM ammonium acetate buffer (pH 5) and incubated for 2 h at 37 °C. The reaction mixtures were precipitated with 200 μL of 0.1% FA in methanol-containing IS (FFA). Samples were vortexed for 5 min at 2000 rpm, and then ethyl acetate was added, the sample was vortexed and centrifuged at 10,000 rpm for 10 min. The organic layer was then collected and dried at 37 °C using a TurboVap and nitrogen stream. The dried residues were reconstituted with mobile phase and injected into LC-MS/MS for analysis.

### 4.7. Validation Parameters

The USFDA and related guidelines were followed for full validation of the LC-MS/MS method of quercetin and *t*-res with several validation parameters including specificity, sensitivity, linearity, recovery, precision, accuracy, and stability [50].

#### 4.7.1. Selectivity and Specificity

Serum samples from six different rats were used to investigate the selectivity by analyzing the extracted drug-free control serum. Specificity was determined by ensuring the absence of chromatographic interference peaks from the serum matrix at retention times of quercetin, *t*-res, and I.S.

#### 4.7.2. Calibration Curve

The concentration of CS was plotted on the X-axis, and the ratio of analyte to I.S was plotted on the Y-axis to develop the calibration curve for quercetin and *t*-res. Concentrations ranging from 5 to 500 (5, 10, 20, 50, 100, 200, and 500 ng/mL) ng/mL were analyzed using least-squares regression with a 1/x^2^ weighing factor. A minimum correlation coefficient (r^2^) value of 0.995 is required. The acceptable range for back-calculated CS was 100 ± 15% of the nominal value, except for LLOQ which was 100 ± 20%.

#### 4.7.3. Accuracy and Precision

To determine the intra-day precision and accuracy, six replicates of four quality control levels were analyzed for each assay. The accuracy and precision of inter-day assays were evaluated by analyzing the QC samples at four levels for three consecutive days. The acceptance criteria must have a standard deviation of ±15%, except LLOQ, which must be within ±20%.

#### 4.7.4. Recovery

The quercetin and *t*-res recovery was determined by comparing the peak area of spiked samples at three QC levels (LQC, MQC, and HQC) before and after extraction.

#### 4.7.5. Matrix Effect

The post-extraction spike method was employed to estimate the matrix effect of quercetin, *t*-res, and I.S. Matrix effect was evaluated across three QC levels (n = 6) LQC, MQC and HQC. The matrix effect was calculated using the comparison of the peak area of the analyte in the post-extracted spiked QC serum sample to the peak area of the analyte with an equivalent concentration in the mobile phase.

#### 4.7.6. Carry over and Dilution Integrity

Carryover was determined by injecting a blank sample after the highest calibration sample. Dilution integrity was evaluated by diluting the 5000 ng/mL of both analytes (n = 6 replicates) to attain 500 ng/mL using the blank serum. The accuracy and precision for dilution integrity experiments should be within ±15%.

#### 4.7.7. Stability Studies

Stability studies were performed using three different QC levels including LQC, MQC, and HQC with six replicates. The extracted serum samples were analyzed using different storage conditions including autosampler (4 ± 2 °C for 24 h), bench top stability (24 ± 4 °C for 8 h), and long-term stability (−70 ± 10 °C for 30 days). Freeze-thaw stability was evaluated after subjecting the samples to three cycles of freezing (−70 ± 10 °C for 12 h) and thawing. Stability samples were quantified against fresh calibration and QC samples. The accuracy and precision of stability samples should meet the acceptance criterion of ±15%.

#### 4.7.8. Stability in Simulated Gastric and Intestinal Fluids

The stability of quercetin and *t*-res was tested at 100 μg/mL in simulated gastric (SGF, pH 1.2) and intestinal (SIF, pH 6.8) fluids (n = 3). The manufacturer’s protocol was followed to prepare biorelevant media with enzymes [51]. Quercetin and *t*-res were exposed to SGF and SIF buffers with and without enzymes and incubated in a shaking water bath at 37 °C. 100 μL samples were collected at specific intervals (0–60 min for SGF and 0–180 min for SIF). At a concentration of 100 µg/mL samples were extracted using ACN containing I.S (1:2, *v*/*v*), vortexed for 5 min, and then centrifuged at 10,000 rpm for 5 min [52]. The supernatant of the samples was utilized for LC-MS/MS analysis. The % remaining of quercetin and *t*-res was calculated to determine the stability using Equation (1).

(1) $$\%\;Drug\;remaining=\frac{Concentration\;of\;drug\;at\;time\;t}{Concentration\;of\;drug\;at\;time\;0}\times 100$$

#### 4.7.9. Plasma Stability

The preincubation of rat plasma was done for 5 min at 37 °C. After pre-incubation, quercetin and *t*-res were added to plasma at a concentration of 100 µg/mL, and reaction tubes were incubated for 3 h in a shaking water bath at 37 °C. Aliquots were collected at predetermined timepoints of 0, 5, 15, 30, 45, 60, 90, 120, and 180 min. Plasma samples were processed immediately using the extraction method mentioned in Section 4.6. The % remaining of the drug at various time intervals relative to the initial time point (time 0) was used to calculate the plasma stability.

#### 4.7.10. Metabolic Stability in Rat Liver Microsomes (RLM)

Microsomal metabolic stability was conducted using in-house prepared RLM and the procedure was reported in the previous publication from our lab [53]. The 0.8 mL reaction mixture was prepared using RLM at 0.5 mg/mL, 40 mM MgCl_2_, in 50 mM Tris-HCl buffer (pH 7.4). 100 µg/mL of quercetin and *t*-res was added to the reaction mixture and preincubated in a shaking water bath at 37 °C and 1 mM NADPH was added to the reaction milieu to initiate the reactions. Samples were collected at predetermined time points up to 60 min and processed for LC-MS/MS analysis [54].

#### 4.7.11. In-Vivo Pharmacokinetic Study

The pharmacokinetic study of quercetin and *t*-res was performed by an oral administration of EECQ extract (400 mg/kg) in male SD rats (n = 6). The EECQ oral dose was selected based on our previous publication [1]. The calculated amounts of quercetin and *t*-res in the 400 mg/kg dose of the extract were found to be (24.33 ± 1.24 µg/mg extract) and (13.99 ± 3.52 µg/mg extract), respectively. Blood samples were collected from the retro-orbital plexus of rats at predetermined time points 0.25, 0.5, 1, 2, 4, 8, 12, 24, and 48 h after oral administration. Serum was obtained by centrifuging the blood and samples were stored at −80 °C until analysis. Serum samples were processed using the LLE extraction mentioned in Section 4.6 and analyzed using the validated LC-MS/MS method [55]. The non-compartment model was used to calculate the pharmacokinetic (PK) parameters for quercetin and *t*-res using WinNonlin 6.3 software (Pharsight Corporation, Saint Louis, MO, USA). The PK parameters were estimated, including C_max_, maximum plasma concentration; t_1/2_, half-life; T_max_, time to reach C_max_; CL/F, clearance and Vd/F, apparent volume of distribution. AUC_0–t_ (area under the concentration curve from zero time point to the last time point) was determined with the trapezoidal rule up to the last measured plasma concentration, C_last_. AUC_0–∞_ (AUC curve from zero time point to infinity) was determined by adding AUC_0–t_ and C_last_/K_e_.

## 5. Conclusions

The developed method has advantages with short analysis run time 5 min, inexpensive LLE sample processing, low serum volume requirements (100 μL), lack of matrix and carryover effects, and high sensitivity. Additionally, the validated method was reproducible. Both Quercetin and *t*-res remained stable during sample processing and under various storage conditions. The tested concentration range in rat serum displayed exceptional linearity for quercetin and *t*-res. The LLE method has good recovery for the extraction of quercetin, *t*-res, and I.S in rat serum. The method was successfully applied to investigate the pharmacokinetics of EECQ extract in a single oral dose. The validated LC-MS/MS method was repeatable and can be applicable to different preclinical and exploratory studies, such as toxicokinetic and clinical PK studies. Our in vivo results suggest that intragastric administration may be a more effective route of administration for quercetin and ***t***-res, as it leads to higher C**_max_**, shorter half-life, and high volume of distribution. Therefore, the development of a validated LC-MS/MS method for the simultaneous estimation of quercetin and *t*-res in *Cissus quadrangularis* extract in rat serum is essential to understand their pharmacokinetic properties and stability. The results of this study will provide important information for the development of *Cissus quadrangularis* extract-based formulations, the therapeutic potential of *Cissus quadrangularis* extract in animal models and possibly in humans for the treatment of various diseases.

## Figures and Tables

**Table 1:** Optimized LC-MS/MS compound and source parameters for EECQ markers (Quercetin and *t-res*) and internal standard.

Compound Parameters	*trans*-Resveratrol	Quercetin	Fenofibric Acid (I.S)
Negative Mode	[M-H]^−1^		
Parent ion Q1 *m*/*z*)	227	301	317
Product ion Q3 (*m*/*z*)	143	151	230.9
DT (ms)	150	150	150
DP (eV)	−98	−105	−53
EP (eV)	−10	−10	−12
CE (eV)	−38	−30	−18
CXP (eV)	−6	−6	−10
Source Parameters: CUR = 25, CAD = Medium, IS = −4500 V, Temp = 400 °C, GS1 and GS2 = 50:50			

**Table 2:** The linear range, LLOQ, regression equations, and correlation coefficient values for the analytes, quercetin, and *t*-res.

Analyte	Linearity Ranges (ng/mL)	LLOQ (ng/mL)	Regression Equation	Correlation Coefficient (r^2^)
Quercetin	5–500	5	0.00336x + 0.0006	≥0.9974
*trans*-resveratrol	5–500	5	0.00242x + 0.00084	≥0.9938

**Table 3:** The intra- and inter-day precision and accuracy for the *quercetin* and *t*-res analytes in rat serum (n = 6).

Analyte	Spiked Conc. (ng/mL)	Intraday Assay			Interday Assay		
		Measured Conc. (ng/mL)	Accuracy (% RE)	Precision (% RSD)	Measured Conc. (ng/mL)	Accuracy (% RE)	Precision (% RSD)
Quercetin	5	5.04 ± 0.45	100.72	8.86	4.89 ± 0.37	97.88	7.52
	30	29.99 ± 1.91	99.97	6.37	30.09 ± 2.62	100.30	8.71
	203	188.33 ± 12.07	92.77	6.41	193.02 ± 18.34	95.08	9.50
	375	372.42 ± 19.04	99.31	5.11	387.40 ± 30.35	103.31	7.84
*trans*-resveratrol	5	5.20 ± 0.29	104.03	5.66	4.68 ± 0.20	93.68	4.35
	30	30.61 ± 1.57	102.04	5.14	28.72 ± 2.76	95.74	9.61
	203	189.03 ± 8.70	93.12	4.60	186.83 ± 12.75	92.03	6.82
	375	392.19 ± 13.01	104.58	3.32	402.73 ± 24.59	107.39	6.11

**Table 4:** Recoveries and matrix effect of quercetin and *t*-res in rat serum at three QC levels. Data are represented as mean ± SD, n = 6.

Analyte	Spiked Conc. (ng/mL)	Extraction Recovery (%, Mean ± SD)	Matrix Effect (%, Mean ± SD)
Quercetin	30	84.52 ± 5.04	101.32 ± 9.62
	203	87.96 ± 3.01	91.76 ± 5.75
	375	87.87 ± 7.35	91.40 ± 6.56
*trans*-resveratrol	30	86.32 ± 10.14	102.32 ± 5.87
	203	80.52 ± 7.53	96.51 ± 5.55
	375	82.35 ± 4.08	106.41 ± 8.00

**Table 5:** Stability data of quercetin and *t*-res in rat serum (n = 6).

	Autosampler Stability (4 ± 2 °C, 24 h)				Benchtop Stability (24 ± 4 °C, 8 h)			Long-Term Stability (−70 ± 10 °C, 30 Days)			Freeze-Thaw Stability (−70 ± 10 °C, 3 Cycles)		
Analyte	Nominal Conc. (ng/mL)	Measured Conc. (ng/mL)	RSD (%)	Accuracy (%)	Measured Conc. (ng/mL)	RSD (%)	Accuracy (%)	Measured Conc. (ng/mL)	RSD (%)	Accuracy (%)	Measured Conc. (ng/mL)	RSD (%)	Accuracy (%)
Quercetin	30	27.77 ± 1.50	5.41	92.56	27.09 ± 1.67	6.17	90.29	30.07 ± 2.06	6.86	100.22	29.44 ± 2.73	9.27	98.12
	203	181.31 ± 8.65	4.77	89.32	184.64 ± 7.16	3.88	90.96	199.71 ± 8.70	4.35	98.38	190.17 ± 8.97	4.72	93.68
	375	372.92 ± 2.21	0.59	99.44	371.12 ± 2.43	0.66	98.96	373.05 ± 10.65	2.86	99.48	369.86 ± 4.43	1.20	98.63
*trans*-resveratrol	30	28.36 ± 2.19	7.71	94.53	28.52 ± 3.05	10.68	95.08	30.50 ± 2.53	8.28	101.68	27.40 ± 4.45	7.43	91.32
	204	190.28 ± 13.20	6.94	93.73	189.53 ± 8.81	4.65	93.36	189.08 ± 14.18	7.50	93.14	189.43 ± 17.82	9.41	93.32
	375	373.15 ± 5.84	1.56	99.51	368.13 ± 12.29	3.34	98.17	366.25 ± 11.38	3.11	97.67	367.92 ± 11.94	3.25	98.11

**Table 6:** Pharmacokinetic parameters of quercetin and *t*-res after oral administration of 400 mg/Kg EECQ. Data are represented as mean ± SD, n = 6.

Parameter (Unit)	Quercetin (in EECQ)	*trans*-Resveratrol (in EECQ)
C_max_ (ng/mL)	57.65 ± 14.28	56.35 ± 13.14
AUC_0–t_ (h*ng/mL)	447.06 ± 47.05	144.83 ± 20.76
AUC_0–∞_ (h*ng/mL)	474.23 ± 51.49	156.57 ± 21.09
T_max_ (h)	1.17 ± 0.41	0.58 ± 0.20
T_1/2_ (h)	4.91 ± 0.69	1.51 ± 0.40
CL/F (L/h/Kg)	851.55 ± 89.69	2593.43 ± 346.71
V_d_/F (L/Kg)	6017.81 ± 940.82	5663.48 ± 1655.09

## Data Availability

Data supporting the reported results will be available with the corresponding author (Jiaur R. Gayen).

## Sample Availability

Sample availability reported results will be available with the corresponding author (Jiaur R. Gayen).
